# Regenerated Sciatic Nerve Axons Stimulated through a Chronically Implanted Macro-Sieve Electrode

**DOI:** 10.3389/fnins.2016.00557

**Published:** 2016-12-08

**Authors:** Matthew R. MacEwan, Erik R. Zellmer, Jesse J. Wheeler, Harold Burton, Daniel W. Moran

**Affiliations:** ^1^Department of Biomedical Engineering, Washington UniversitySt. Louis, MO, USA; ^2^Department of Neuroscience, Washington University School of Medicine in St. LouisSt. Louis, MO, USA

**Keywords:** peripheral nerve, sieve electrode, peripheral nerve interface, functional electrical stimulation, nerve regeneration

## Abstract

Sieve electrodes provide a chronic interface for stimulating peripheral nerve axons. Yet, successful utilization requires robust axonal regeneration through the implanted electrode. The present study determined the effect of large transit zones in enhancing axonal regeneration and revealed an intimate neural interface with an implanted sieve electrode. Fabrication of the polyimide sieve electrodes employed sacrificial photolithography. The manufactured macro-sieve electrode (MSE) contained nine large transit zones with areas of ~0.285 mm^2^ surrounded by eight Pt-Ir metallized electrode sites. Prior to implantation, saline, or glial derived neurotropic factor (GDNF) was injected into nerve guidance silicone-conduits with or without a MSE. The MSE assembly or a nerve guidance conduit was implanted between transected ends of the sciatic nerve in adult male Lewis rats. At 3 months post-operation, fiber counts were similar through both implant types. Likewise, stimulation of nerves regenerated through a MSE or an open silicone conduit evoked comparable muscle forces. These results showed that nerve regeneration was comparable through MSE transit zones and an open conduit. GDNF had a minimal positive effect on the quality and morphology of fibers regenerating through the MSE; thus, the MSE may reduce reliance on GDNF to augment axonal regeneration. Selective stimulation of several individual muscles was achieved through monopolar stimulation of individual electrodes sites suggesting that the MSE might be an optimal platform for functional neuromuscular stimulation.

## Introduction

Peripheral nerves are a common biological target for neuroprosthetic devices (Navarro et al., 2005). In particular, electrical stimulation of motor axons with chronically-implanted neural interfaces provides a method of controlling distal musculature and restoring motor function following injury. Compared to functional electrical stimulation of muscle tissue, stimulation of peripheral nerves requires lower excitation thresholds and enables recruitment of multiple distinct muscles with a single implanted electrode (Wei and Grill, 2009).

Existing neural interfaces consist of both extraneural and intraneural electrodes; each possesses distinct benefits and limitations. Extraneural devices include cuff electrodes (Veraart et al., 1993; Loeb and Peck, 1996), slowly penetrating interfascicular nerve electrodes (SPINEs; Tyler and Durand, 1997), and flat-interface nerve electrodes (FINEs; Tyler and Durand, 2002). Extraneural devices are easy to implant, robust, and minimally destructive. However, lacking close contact with nerve fibers or fascicles, they require high excitation current thresholds and have limited recruitment specificity. Intraneural devices include the Utah electrode array (UEA), Utah slanted electrode array (USEA; Normann, 2007), and longitudinally implanted intrafascicular electrodes (LIFEs; Lawrence et al., 2003). All make intimate contact with interfaced nerves, which results in low excitation thresholds and high recruitment specificity (Yoshida and Horch, 1993; Branner et al., 2001; McDonnall et al., 2004a,b). Yet, difficulty of electrode insertion as well as the mismatch in mechanical compliance between nerve and electrode can result in damage to the target nerve and reduce long-term efficacy (Branner et al., 2004).

Sieve electrodes offer an alternative to existing peripheral nerve interfaces. By design, sieve electrodes contain transit zones through which regenerating axons can grow, thereby integrating the device into the native peripheral nerve tissue (Navarro et al., 2005). Implantation requires transecting the target nerve and placing a thin sieve electrode between surgically opposed proximal and distal nerve stumps. Axons regenerating from the proximal nerve stump pass through transit zones in the sieve electrode and extend into the distal nerve stump. These axons can be electrically stimulated from metalized electrode sites at the edges of the transit zones in the sieve electrode.

Prior sieve electrodes had transit zones with hundreds of small holes (10–60 μm diameter) that facilitated recording from small groups of peripheral axons (Kovacs et al., 1992). Optimization of these silicon and polymeric micro-sieve electrodes aimed at improving axonal regeneration (Akin et al., 1994; Stieglitz et al., 1997; Zhao et al., 1997; Wallman et al., 2001). Axonal regeneration through these micro-sieve electrode arrays was robust in a rat sciatic nerve model (Lago et al., 2007). However, distal motor fibers received poor re-innervation from myelinated fibers due to axonal constriction resulting in focal demyelination at the sieve interface (Lago et al., 2007). Consequently, preservation of muscle mass and muscle force output distal to the site of micro-sieve electrode implantation was significantly impaired. The failure of prior micro-sieve electrodes to support functional motor recovery post-implantation significantly limited motor restoration through functional nerve stimulation.

The present study examined a novel “macro-sieve” electrode, its design, fabrication, implantation and evaluation in an *in vivo* rat sciatic nerve model, both with and without neurotrophic support from Glial-Derived Neurotropic factor (GDNF; Wood et al., 2009, 2011). The target nerve was exposed to GDNF as previously demonstrated *in vivo* (Sakiyama-Elbert and Hubbell, 2000a,b; Sakiyama-Elbert et al., 2001; Lee et al., 2003). Histological comparisons across experimental groups examined regenerated fibers proximal and distal to the electrode, as well as within transit zones. Electrophysiological evaluation of regenerated nerve fibers included determining motor axon compound action potentials and activation levels of distal muscles. Additionally, behavioral assessment examined whether functional recovery resulting from muscle re-innervation supported motor function and dexterity.

## Methods

### Experimental design

Forty adult male Lewis rats (275–300 g) were randomized into five experimental groups of eight animals each (Table 1). Group I were controls not exposed to surgery. Groups II–V underwent surgical transection of the sciatic nerve followed by microsurgical implants of nerve guidance conduits made of silicone with or without the manufactured macro-sieve electrode (MSE). Groups II and III had silicone conduits and, respectively, received sterile saline or a fibrin-based delivery system loaded with GDNF. Groups IV and V had the MSE assembly and, respectively, received sterile saline or GDNF. All groups underwent evaluation of sciatic nerve function upon termination of the study 3 months post-surgery. Histological analyses examined the morphology of sciatic nerves from all cases after excision and fixation. All experimental procedures were in strict accordance with regulations set forth by the Animal Studies Committee and the Division of Comparative Medicine at Washington University.

**Table 1:** **Experimental design**.

**Group**	**I. Control**	**II. Conduit**	**III. Conduit**	**IV. Sieve**	**V. Sieve**
Electrode	None	None	None	Macro-sieve	Macro-sieve
Conduit	None	Silicone conduit	Silicone conduit	Silicone conduit	Silicone conduit
Regen. System	None	Saline	Fibrin DS + 100 ng/ml GDNF	Saline	Fibrin DS + 100 ng/ml GDNF
	*n* = 8	*n* = 8	*n* = 8	*n* = 8	*n* = 8

*Experimental groups utilized in the investigation of MSEs with and without GDNF-loaded delivery system. DS, delivery system; GDNF, glial derived neurotropic factor*.

### Fabrication of macro-sieve electrodes

**Figure 1:**
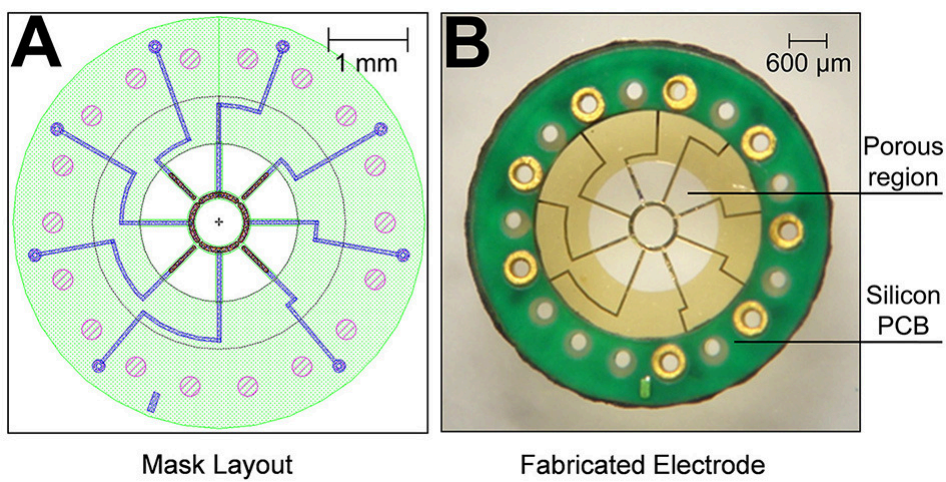
**Fabricated macro-sieve electrode. (A)** Macro-sieve schematic showing polyimide base (green), gold traces (blue), and platinized electrode sites (speckled red-black). **(B)** Optical micrograph of MSE with ultrasonically-bonded micro PCB connector.

**Figure 2:**
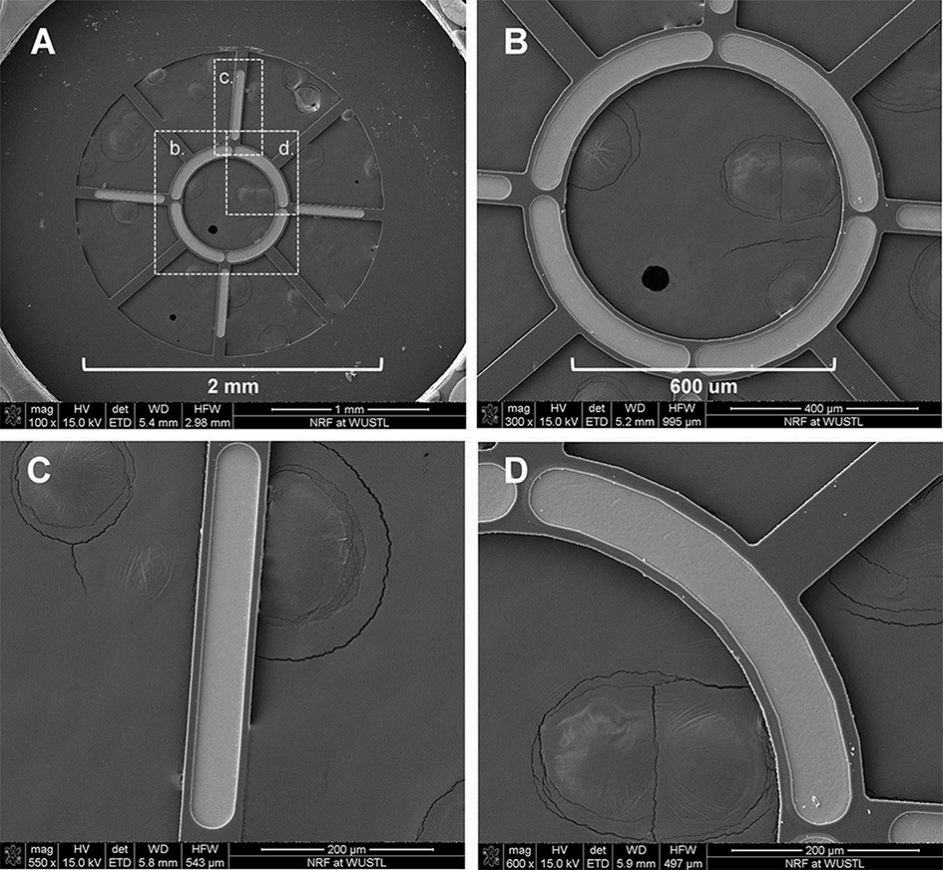
**Scanning electron micrographs of macro-sieve electrodes. (A)** Scanning electron micrographs demonstrate the features of the porous region of MSEs. Dashed boxes with labels b, c, and d were magnified in **(B–D)**, respectively.

The MSEs had a central porous region 2 mm in diameter containing nine transit zones. The central transit zone was a circular region of ~600 μm diameter surrounded by eight rhomboid shaped regions (Figure 1B, light gray region; Figure 2A). Despite shape differences, the central and peripheral transit zones had comparable cross sectional areas of ~0.285 mm^2^. A concentric pattern of eight radiating traces and peripheral connector pads (Figures 1A,B) allowed connection to the Pt-Ir electrode sites that border the transit zones. Four circumferential electrode sites surrounded the central transit zone, allowing electrical activation of different sectors within the cross section of the regenerated nerve (Figures 2B,D). Likewise, four straight peripheral electrode sites were located on alternating spokes between neighboring peripheral transit zones (Figures 2A,C). Using current steering techniques, the eight Pt-Ir electrode sites in the MSE could allow targeting of smaller precise sectors of axons within all nine transit zones.

Peripheral connector pads expedited ultrasonic bonding of the polyimide MSEs to a circular, micro-printed circuit board (I.D. = 3.2 mm, O.D. = 5.2 mm). Microwire leads were soldered onto micro-printed circuit boards to facilitate an electrical interface between metallized sites on the polyimide electrode with external instrumentation. The construction of electrode substrate and insulation material was achieved with polyimide resins and custom manufactured for this project by NeuroNexus Inc., (Ann Arbor, MI).

### Macro-sieve electrode assemblies

**Figure 3:**
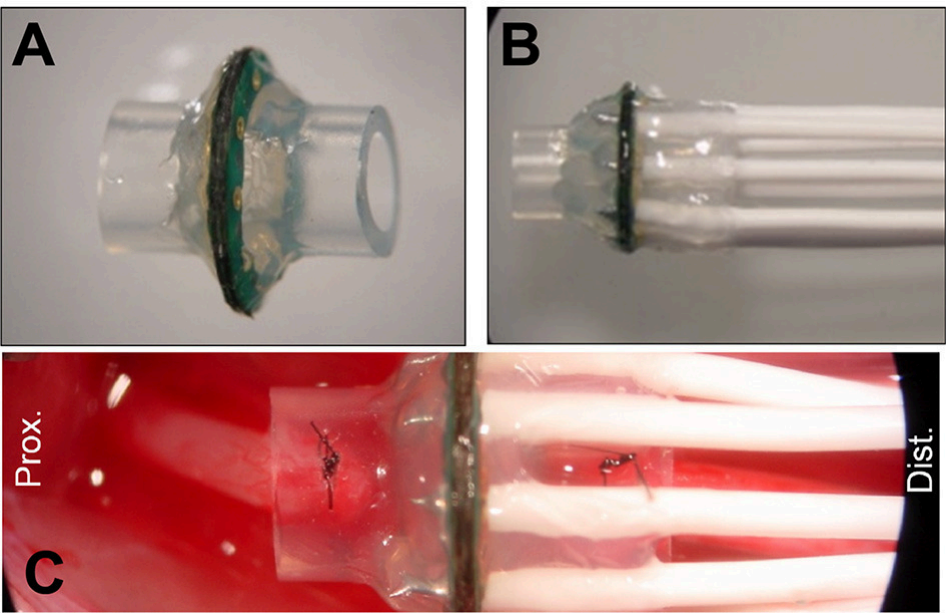
**Macro-sieve electrode assembly and high magnification view of implanted macro-sieve electrode. (A)** Electrode with attached silicone nerve guidance. **(B)** Completed sieve electrode assembly with microwire leads encapsulated in silicone elastomer. **(C)** Intra-operation photograph of a MSE assembly implanted between the transected ends of the rat sciatic nerve.

Micro-wire leads connected to metallized contact sites on the electrodes were soldered to a female Omnetics 20-pin connector. MSEs were then embedded in medical grade epoxy (EpoTek 730, Epoxy Technologies Inc., Billerica, MA) for protection of the electrode assembly and for insulating connections between the polyimide electrode and micro-circuit board. Prior to chronic implantation, silicone nerve guidance conduits (length = 3 mm, I.D. = 2 mm, O.D. = 3.2 mm, A-M Systems Inc., Carlsborg, WA) were attached to both sides of the MSE, forming an assembly. All MSE assemblies were finally encapsulated in 1–2 mm of medical grade elastomer (MDX4–4210, Dow Corning, Midland, MI; Figures 3A,B) to provide mechanical support. Nerve guidance conduits directed axonal regeneration through the electrode and between the proximal and distal nerve stumps (Figure 3C). Silicone nerve guidance conduits (length = 6 mm) without sieve electrodes were used as controls.

### Fibrin-based delivery system

Fibrinogen solutions (8 mg/ml) were prepared by dissolving human plasminogen-free fibrinogen (Calbiotech Inc., Spring Valley, CA) in deionized water for 1 h at 37°C, then dialyzing the solution against 4 L of Tris-buffered saline (TBS) containing 33 mM Tris, 8 g/L NaCl, and 0.2 g/L KCl (Sigma-Aldrich, St. Louis, MO)^1^. The fibrinogen solution was sterile filtered using 5.0 and 0.22 μm syringe filters; spectrophotometry confirmed the concentration of fibrinogen. Just prior to surgery, fibrinogen, TBS, 50 mM CaCl_2_ in TBS, 20 U/ml thrombin, 25 mg/ml a_2_PI_1–7_-ATIII_124–134_ peptide, 45 mg/ml heparin, and 100 ng/ml human GDNF (Peprotech, Rocky Hill, NJ) were mixed in an Eppendorf tube (Lee et al., 2003). Immediately after mixing, 30 μl of the fibrin-based delivery system containing GDNF was injected into the nerve guidance conduits of Groups III and V. Prior to surgical implantation, applied fibrin matrices polymerized for 5 min.

### Surgical procedures

Animals were anesthetized by inhalation using 4% Isoflurane (induction) and 2% Isoflurane (maintenance). All procedures were conducted with aseptic techniques. Survival surgery involved transection of the sciatic nerve; proximal and distal nerve stumps were secured inside silicone guidance conduits with or without MSEs leaving a gap of ~4 mm. Microwire leads were routed under proximal musculature and into a subcutaneous pocket on the back of the animal. Omnetics connectors were implanted subcutaneously for re-exposure and terminal nerve interfacing. Layered closure of muscle and skin proceeded, respectively, using 5-0 vicryl and 4-0 nylon suture. All animals wore a polyethylene collar for 1 week post-operatively to prevent autophagia and disruption of the wound.

The same anesthesia protocol was used for non-survival surgery at 3 months. The sciatic nerve and distal musculature were exposed for assessment of nerve conduction and re-innervated muscle physiology during this terminal stage.

### Gross morphology of regenerated axons

Excised and fixed nerve segments from all experimental groups were examined and photographed using a stereo microscope (Olympus). Regenerated nerve segments contained in MSE assemblies were removed without disturbing the transit zones of the implanted sieve electrode.

### Histomorphometric analysis of regenerated nerve

Distal and proximal nerve segments and intervening MSE assemblies (*n* = 8) from each experimental group were excised and fixed by submersion in cold 3% gluteraldehyde in 0.1 M phosphate buffer (pH = 7.2). Nerve tissue was dissected from the silicone conduit segments and the transit zones in the MSEs, dehydrated in ethanol, post-fixed with 1% osmium tetroxide, and embedded in Araldite 502 epoxy resin (Polysciences, Warrington, PA). Cross sections <1 μm thick were cut with an LKB III Ultramicrotome (LKB-Produkter A.B., Bromma, Sweden) 5 mm proximal, immediately proximal, immediately distal, and 5 mm distal to the integrated electrode and stained with 1% toluidine blue. A blinded observer analyzed and scored all slides.

Quantitative analysis was performed on toluidine blue stained sections using a semi-automated digital image analysis system, linked to software (LECO Instruments, St. Joseph, MI), as previously described (Hunter et al., 2007). Primary metrics obtained from digitized 1000X magnified fields included the number, density, percent nerve tissue (100 × neural area/intrafascicular area), mean fiber width (μm), myelinated fiber area (μm^2^), and myelin area (μm^2^).

### Electrophysiological assessment of nerve function

#### Compound neural action potential

The right sciatic nerve and its branches were isolated from the sciatic notch to below the point of trifurcation, distal to the popliteal artery. Bipolar silver wire electrodes, placed on the sciatic nerve proximal to the implant site, delivered cathodic monophasic electrical stimuli. Similar electrodes on the regenerated segment distal to the implant site in the peroneal nerve provided differential recordings of propagating compound neural action potentials (CNAPs). Amplified CNAPs were bandpass filtered (LP = 1 Hz, HP = 5 kHz, notch 60 Hz) and recorded on a desktop PC.

Software synchronized stimulation and recording of induced CNAPs such that electrical stimulation coincided with the initiation of a 20 ms recording period, with data sampled at 40 kHz. The mean CNAP response was an average from 25 trials taken at tested stimulation amplitudes. The latter increased incrementally until peak-to-peak amplitude of the mean CNAP response stabilized. Absence of retrograde stimulation of spinal motor reflexes was confirmed by repeating CNAP recording at the optimal stimulus amplitude after crushing the sciatic nerve at the sciatic notch for 30 s using No. 5 Jeweler's forceps. Intact sciatic and peroneal nerves were similarly tested in control animals.

#### Electromyography

Trains of monophasic electrical stimuli were applied to the rat sciatic nerve proximal to the implant site while electromyograms (EMGs) were recorded differentially from reinnervated distal *tibialis anterior* (TA), *gastrocnemius* (G), and *extensor digitorum longus* (EDL) muscles using intramuscular microwire electrodes. Software synchronized stimulation and recording in a 500 ms recording window, with data sampled at 10 kHz. The raw data was rectified and applied to a peak detector that averaged results across trains of stimulation, yielding a measure of evoked EMG responses (essentially a ½ peak-to-peak average). Similar testing was performed in control animals.

#### Evoked muscle force measurement

The force of evoked motor responses in distal musculature was assessed at 3 months post-surgery. Evoked force production was measured in EDL, TA, and G muscles. The right sciatic nerve and its branches were isolated from the sciatic notch to below the point of trifurcation, distal to the popliteal artery. Additionally, EDL, TA, and G were exposed through skin incisions extending along the anterior and posterior portion of the lower leg. Following isolation, the distal portions of the EDL, TA, and G muscles were separated from the leg by severing the distal tendons. In order to obtain force measurements, animals were immobilized by anchoring the femoral condyles to a custom force jig and distal tendons were attached to thin film load cells (S100, Strain Measurement Devices Inc., Meriden, CT). The load cells transduced evoked muscle force responses to electrical stimulation of the macro sieve electrode sites as well as silver wire electrodes placed on the sciatic nerve proximal to the MSE assembly. Recorded signals were amplified (gain = 1000X) using instrumentation amplifiers (AD620, Analog Devices Inc., Norwood, MA) powered by a constant voltage source before being recorded on a desktop PC equipped with a data acquisition board and custom Matlab software. As noted above, synchronization of stimulation and recording coincided with the initiation of a 500 ms recording period, with data sampled at 4 kHz. Software calculated the passive and active forces for each recorded trace. Individual stimulus pulses (duration 50–200 μs, amplitude 0–1 mA) were routed to either individual MSE electrode sites, or all eight MSE electrode sites simultaneously.

Twitch contraction measurements were utilized to determine the optimal stimulus amplitude (V_o_) and muscle length (L_o_) for isometric force production in each muscle (Yoshimura et al., 1999). Stimulus amplitudes increased incrementally while holding individual muscle length constant. V_o_ was defined as the stimulus amplitude at which the largest active force was produced. Individual muscle length then increased in 1 mm increments from a relaxed state while the stimulation amplitude remained constant at V_o_. Upon determining the muscle length at which the largest active twitch force was produced, a single 300 ms burst of impulses (frequency = 80 Hz) was used to stimulate the sciatic nerve after which muscle length was re-evaluated. L_o_ was directly measured as the length of target muscle from proximal to distal musculotendinous junction. V_o_ and L_o_ were subsequently used for all isometric force measurements including single twitch peak and twitch force (F_t_).

### Analysis of functional motor recovery

#### Walking track analysis

Gait was analyzed at 1, 2, and 3 months post-operation using an 8 × 40 cm walking track lined with paper (Bain et al., 1989; Brown et al., 1989). Red and blue ink, applied to the right and left hind feet of each animal, respectively, traced foot placement and gait during ambulation. A custom-MATLAB program analyzed scanned and digitized marked paper strips and calculated a sciatic function index (SFI) (de Medinaceli et al., 1982; Bain et al., 1989) using a custom graphic interface. The SFI formula Bain proposed was designed to noninvasively track regeneration following nerve crush or transection. It is a metric to quantify sciatic nerve damage that is acquired through walking track analysis. The SFI measured from healthy control animals and animals having recently undergone complete transection of the sciatic nerve are expected, respectively, to be approximately 0 and −100 (Bain et al., 1989).

#### Grid-grip analysis

Grid-grip analysis was performed 3 months post-surgery as a measure of motor recovery in the hindlimb (Metz et al., 2000; Wood et al., 2009, Johnson et al., 2010). Blinded examiners observed each animal walking for a period of 3 min on a fixed grid of bars containing 1.5″ × 1.5″ gaps, and scored individual animals on the number of successful grips of the grid. The definition of a successful grip consisted of a hind limb placement wherein two or more toes of the operated foot gripped the bar to facilitate movement to another bar without slipping. Data was the percent of successful grips made by the operated foot. Animals from all groups (Table 1) had to make at least 20 steps in order to account for variability in the activity level and mobility of individual animals.

#### Maximum angle of ankle extension

The maximum angle of ankle plantarflexion was measured in the operated limb at 3 months post-surgery. Passive analysis of ankle plantarflexion served as an evaluation of maximum range of motion and degree of contraction following implantation of nerve guidance conduits with and without MSEs. Measurements of the maximal angle achieved between the tibia and the metatarsus was with the animal placed in a prone position.

## Results

### Macro-sieve electrode fabrication and assembly

Figures 1A,B show the mask layout and fabricated MSE produced by sacrificial photolithography. Scanning electron micrographs revealed well-defined features and electrode sites (Figure 2B) in the MSE. The central curved electrode sites have a surface area of ~33,000 μm^2^ while the peripheral straight sites on the spokes has a surface area of ~24,000 μm^2^. There was no overlap in metallization between sites (Figures 2B,D). Individual platinized electrode sites demonstrated, at 1 kHz, impedances ranging between 2.3 and 6.3 kΩ with an average of 4.2 ± 0.9 kΩ. All tested metallized sites were functional at the time of implantation.

Bonding the microcircuit board to the polyimide macro-sieve wafer significantly increased the rigidity of the implantable electrode. Bonding the microcircuit board to the polyimide macro-sieve wafer also reinforced electrical connections to the MSE and simplified external connections to micro-wire leads.

### Integration of the macro-sieve electrodes into peripheral nerve tissue

Implanted MSE assemblies and silicone nerve guidance conduits survived *in vivo* throughout the duration of the study without damage or failure in all implanted animals. MSEs maintained proper orientation and placement *in vivo* with the MSE oriented perpendicular to the sciatic nerve.

**Figure 4:**
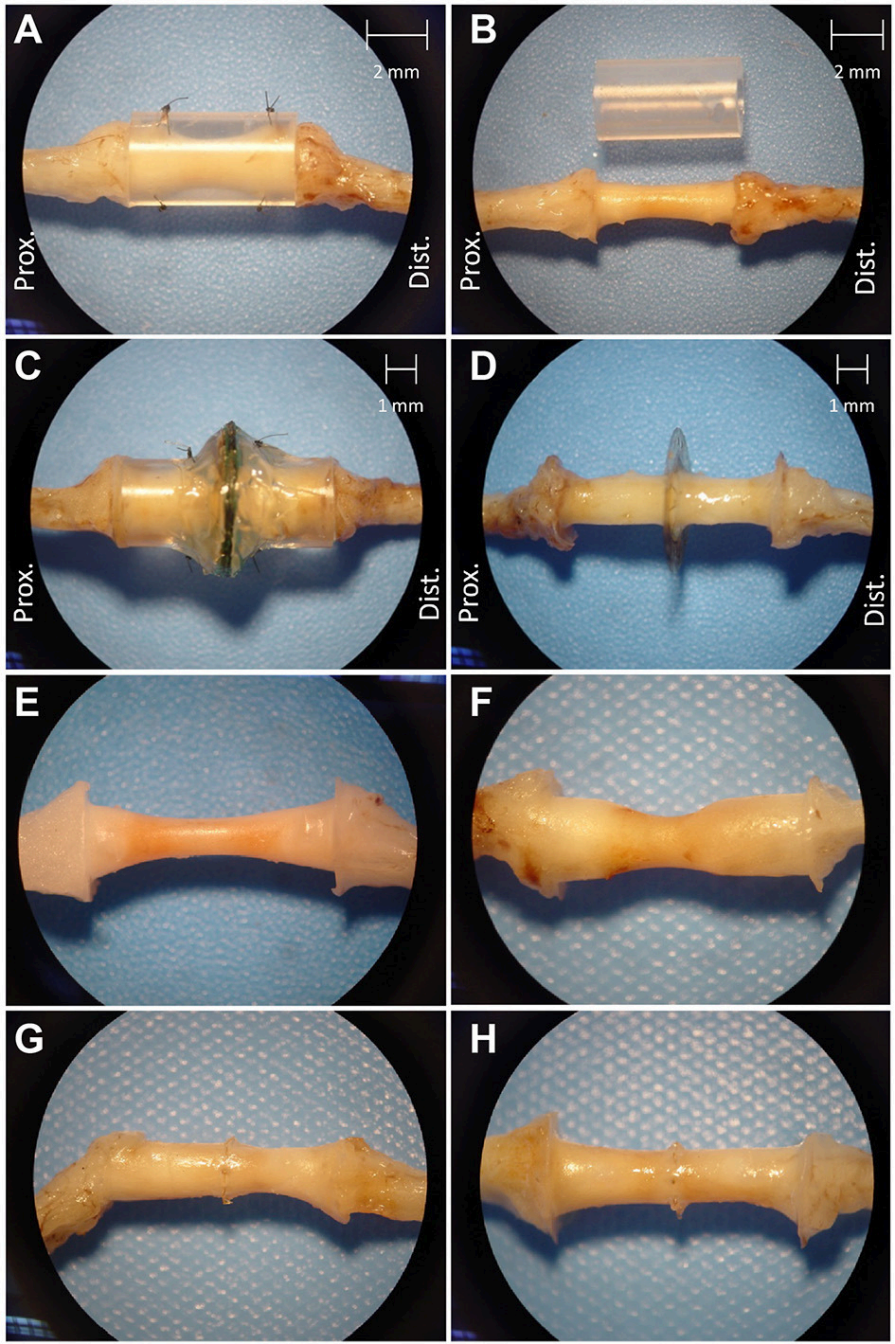
**Excised regenerated sciatic nerve at 3 months post-operation**. Photos of silicone nerve guidance conduits present **(A)** and removed **(B)** from regenerated sciatic nerve without GDNF. Photos of MSE with conduit present **(C)** and removed **(D)**. Photos of regenerated nerve tissue within identical nerve guidance conduits without GDNF **(E)** and with GDNF **(F)**, and in MSEs without **(G)**, and with **(H)** GDNF.

Figure 4 shows that regenerated nerve tissue present within silicone conduits and MSE assemblies had grossly similar singular morphologies that bridged the transected nerve stumps. Regenerated nerve tissue present inside silicon nerve conduits had a “tapered” morphology with a smaller diameter of ~1 mm in the middle of the conduit (Figure 4B, see 2 mm scale). The diameter of the nerve in the middle of nerve guidance conduits was greater with GDNF neurotrophic support (Figure 4E vs. Figure 4F, see 2 mm scale in Figure 4B). Tapering possibly resulted from poor adhesion of regenerated nerve tissue and associated extracellular matrix to the inner lumen of the conduit. Nerves crossing the MSEs did not show a tapered morphology. Regenerated nerve fibers filled the majority of space in the inner lumen of guidance conduits attached to MSEs even without GDNF (Figures 4G,H). This suggests the neurotrophic influence of GDNF was less critical with more accessible transit zones within the MSE.

### Histology of regenerated nerve tissue

**Figure 5:**
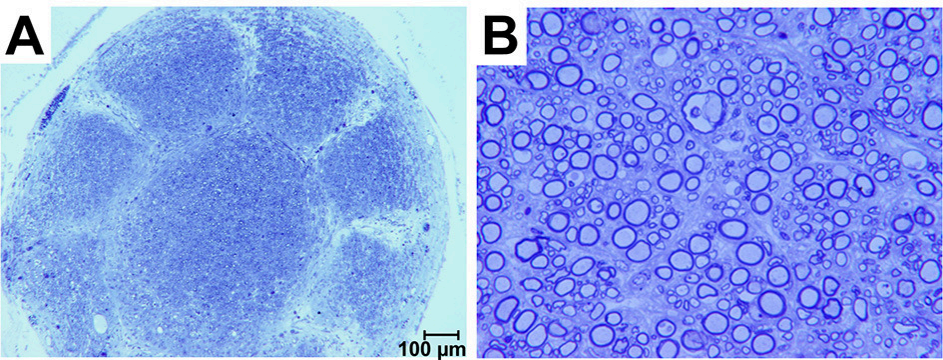
**Photomicrographs of toluidine blue stained axons regenerated in a macro-sieve electrode without GDNF. (A)** Low magnification of a cross-section sliced 300 μm proximal to macro-sieve interface. **(B)** High magnification image shows both myelinated and unmyelinated regenerated axons.

Sections obtained ~300 μm proximal to the interface between the sieve and nerve revealed an architecture mirroring the geometry and organization of the nine transit zones in the electrode. Specifically, one nerve bundle was present per transit zone (Figure 5A). Sections obtained nearer the sieve-nerve interface showed a progressive development and separation of bundles. Toluidine blue-stained sections, taken from just proximal to a transit zone, showed myelinated and unmyelinated peripheral nerve fibers (Figure 5B).

There were no morphometric differences in regenerated axons in the presence or absence of GDNF neurotrophic support. Both preparations had similar distributions of fiber diameters, mean fiber diameters, axon to myelin ratios and organization that mimicked the pattern of transit zones in the electrode. A one-way ANOVA of fiber counts per group was significant (*F* = 19.93, *df* = 2, 23, *p* < 0.0001) probably resulting from significantly (*p* < 0.0001) lower counts in controls (7664 ± 486.4) compared to those in conduits (23,832 ± 4305.6) and MSEs (19,305.6 ± 3527.8) in Bonferroni corrected *t*-tests. Fiber counts also were significantly higher in conduits compared to MSEs (*p* < 0.05).

### Evoked action potential propagation in regenerated nerve tissue

Action potentials evoked from stimulating the proximal end of the nerve propagated through the MSE to recordings from the distal nerve segment. The CNAPs appeared as balanced, biphasic waveforms in regenerated distal sciatic nerve segments with MSEs and with silicone nerve conduits irrespective of GDNF presence. These results suggest that transit zones in the MSE were as conducive to regeneration as open nerve conduits without requiring adjunctive support from GDNF. A one-way ANOVA of peak-to-peak amplitudes was significant (*F* = 23.5, *df* = 4, 39, *p* < 0.0001) mainly because they were significantly larger (*p* < 0.001) in un-operated control nerves (8.44 ± 1.43 mV) than, respectively, in conduits and MSEs with or without GDNF (2.97 ± 1.85 mV; 1.74 ± 1.04 mV; 4.37 ± 1.80 mV; 2.78 ± 1.38 mV) in Bonferroni's multiple comparison tests. Only amplitudes from MSEs with GDNF were significantly larger (*p* < 0.05) than in conduits without GDNF.

### Electrical activation of re-innervated musculature

**Figure 6:**
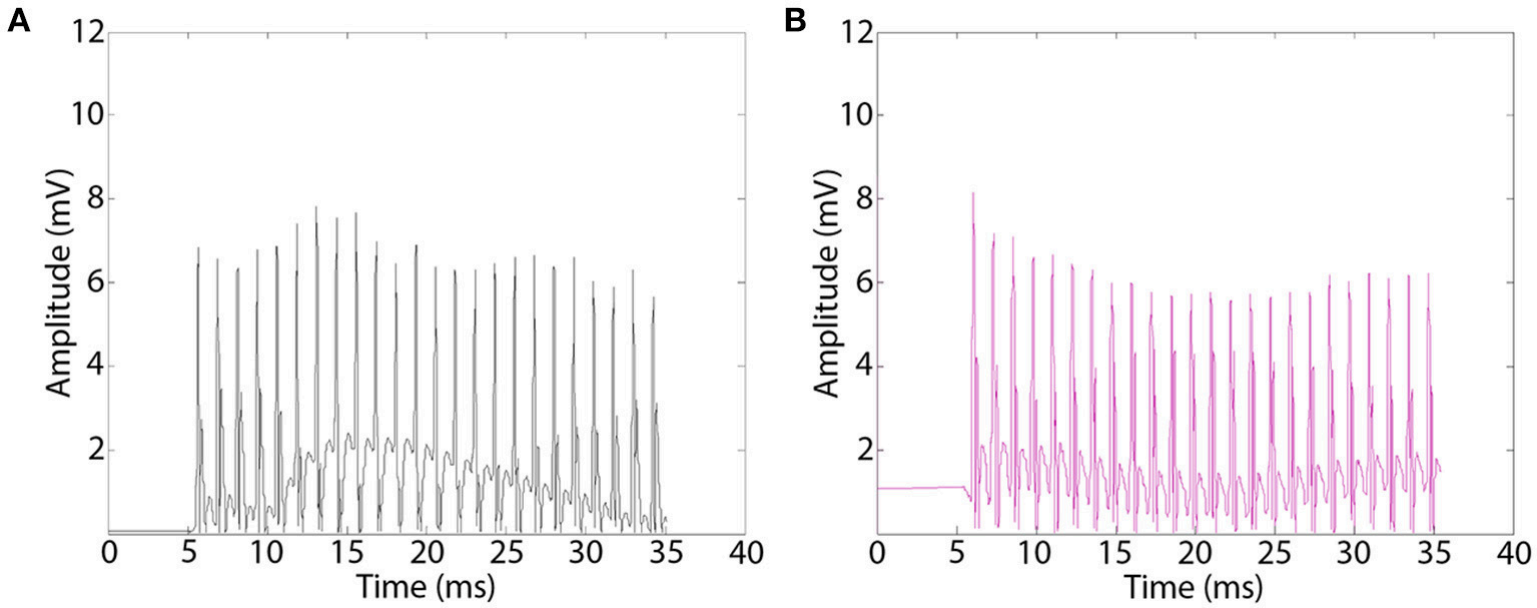
**Representative electromyograms (EMGs) recorded distal to implanted macro-sieve electrode assemblies**. EMGs were evoked using silver wire electrodes placed proximal to implanted devices and recorded distally in the EDL muscle. EMGs successfully measured distal to MSE assemblies with GDNF **(A)**, and nerve conduits with GDNF **(B)**.

Electrical stimulation of the sciatic nerve proximal to the implants by epineurial hook electrodes evoked EMG responses distally in re-innervated musculature. Representative EMGs appeared as biphasic waveforms that were rectified for quantitative offline analysis (Figure 6). EMG recordings showed effective muscle activation in all MSE and silicon nerve conduit implants with and without GDNF, indicating successful regeneration of motor axons. EMGs recorded in EDL distal to MSEs and silicone guidance conduits had comparable amplitudes with no observed effects of GNDF. A one-way ANOVA of EMG amplitudes was not significant. In the presence or absence of GNDF, respectively, amplitudes were 6.06 ± 2.01 and 6.38 ± 2.87 mV in MSEs and were 6.95 ± 1.88 and 5.49 ± 1.87 mV in silicone guidance conduits. Amplitudes in controls were significantly higher (>9 mV). EMG amplitude results from TA and G muscles were similar.

Regenerated nerve segments integrated into MSE assemblies were also stimulated using electrode sites in the MSEs while simultaneously recording evoked muscle force. Three-quarters of the cases with implanted MSEs effectively recruited distal musculature irrespective of GDNF presence. Recruitment of distal musculature required lower current amplitudes from embedded MSEs compared to epineurial hook electrodes. Monopolar stimulation of MSEs with and without GDNF, respectively, exhibited a mean threshold for muscle force activation of 48 ± 32 and 56 ± 28 μA. Comparable activation of muscle force required 173 ± 61 μA with epineurial hook electrodes.

**Figure 7:**
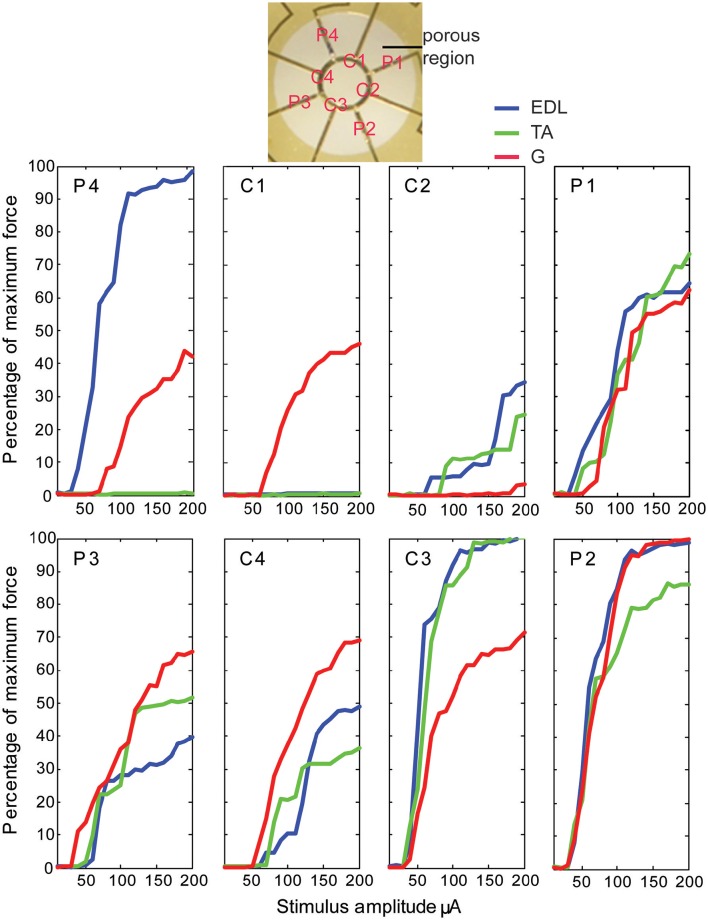
**Motor responses to monopolar stimulation of peripheral nerve tissue through a macro-sieve electrode**. Results from a single case show recruitment curves generated with progressively higher monopolar stimulation currents at each of the eight electrode sites labeled in the image of the porous zone in a MSE. There were 4 peripheral (P) and 4 central (C) metallized sites bordering, respectively, 4 pairs of rhomboid and 4 quadrants of a single circular transit zone. Electrical stimulation evoked force production in *extensor digitorum longus* (EDL), *tibialis anterior* (TA), and *gastrocnemius* (G) muscles.

**Figure 8:**
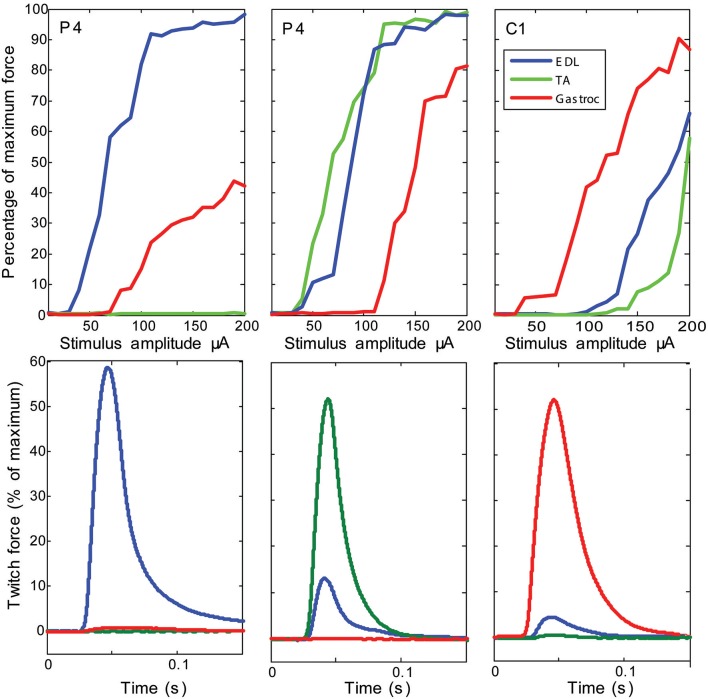
**Examples of maximal selectivity of muscle activation achieved upon monopolar stimulation of peripheral nerve tissue through macro-sieve electrodes**. Data selected from individual cases (each column is a different subject) that highlight achievable selectivity with the MSE upon monopolar stimulation from an electrode site (identified on insert image in Figure 7). The recruitment curves and simultaneous recording of evoked twitch force production in *extensor digitorum longus* (EDL), *tibialis anterior* (TA), and *gastrocnemius* (G) muscles indicate lowest current thresholds and maximal activity from single muscles. Notice that stimulation of the same electrode site in different subjects (site P4 in columns 1 and 2) yields different selectivity.

Stimulation of individual electrode sites on implanted MSEs resulted in minute twitch responses in selective muscle groups at low stimulus amplitudes. Monopolar stimulation of metalized sites for different transit zones varied in efficacy for the three tested muscles (EDL, TA, and G) and in the recruitment of evoked twitch force at different stimulus currents. Plots from an example case (Figure 7) show that the peripheral transit zone site 4 (P4) could selectively activate EDL up to 50% of maximum force with a relatively low threshold stimulation. The central transit zone site 1 (C1) could selectively activate G up to 40% of maximum force. Stimulation of other sites evoked various patterns of muscle activation. However, examples from other cases show that monopolar stimulation can elicit maximal twitch responses for single muscles at selected current thresholds (Figure 8). Muscle specificity exhibited by individual electrode sites was eliminated upon increasing stimulus amplitude or simultaneous stimulation at multiple electrode sites. Neither epineurial stimulation of regenerated nerve tissue nor from MSE sites elicited force levels equal to that in controls.

### Functional motor recovery

**Figure 9:**
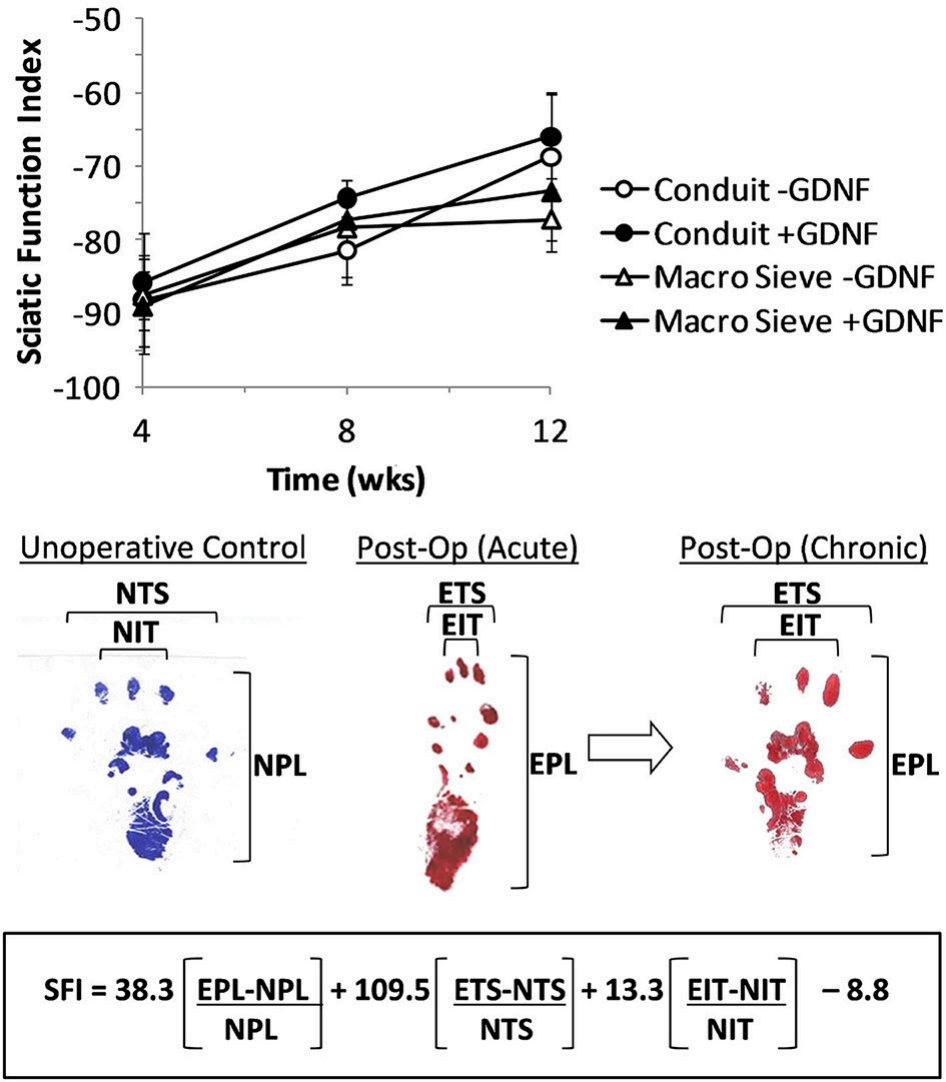
**Calculation of sciatic function index (SFI) from walking track analysis**. SFI measurements over 3 months of implantation (top). Shown are representative hind limb prints for controls (left panel), acutely after nerve transection (middle panel), and after chronic recovery (right panel). Multipliers for each factor and final subtraction based on previously published values (Bain et al., 1989). EPL, experimental print length; NPL, normal print length; ETS, experimental toe spread; NTS, normal toe spread; EIT, experimental intermediary toe spread; NIT, normal intermediary toe spread.

Walking improved over the 3-month post-surgery interval. Representative foot tracking plots showed progressive increase in toe spread and decrease in print length from the acute to chronic post-op measurements (Figure 9). The latter more closely resembled tracks from a control case. Increased SFI scores from −90 to − 70 quantified improved walking over 3 months post-surgery. Behavioral improvements occurred with both implant types and with no significant differences attributable to the presence of GDNF. The outcomes suggest successful re-innervation of distal musculature following implantation of MSEs or nerve guidance conduits.

Grid grip analysis demonstrated sensorimotor deficits consistent with nerve transection and subsequent sciatic nerve regeneration. Tested animals had 7.6–11.5% successful grips, highlighting the residual motor deficit experienced following nerve transection and repair. A one-way ANOVA of percent successful grips showed no significant differences in grid grip performance across experimental groups. Percent successful grips were 11.5 ± 2.6, 7.6 ± 3.0, 9.1 ± 2.2, and 8.5 ± 3.3%, respectively, for MSEs and nerve guidance conduits with and without GDNF.

Ankle plantarflexion was more limited in experimental groups compared to controls, but similar between matched experimental groups per implant type. A one-way ANOVA of maximum degrees of ankle flexion was significant (*F* = 20.05, df = 4, 39, *p* < 0.0001) mainly because of significantly greater flexion (*p* < 0.001) in un-operated control nerves (155 ± 7.58°) than, respectively, with or without GDNF in conduits (120 ± 5°; 103 ± 13°) and MSEs (125 ± 21°; 109 ± 11°) in Bonferroni's multiple comparison tests. These results further confirm that MSEs had a negligible effect on nerve regeneration and in re-establishing terminal motor function compared to empty silicone guidance conduits.

## Discussion

### Viability of the macro-sieve electrode design

Several design features optimized the fabrication of MSEs. Commercial-grade processes and masks produced high yields and consistent assemblies. The design of the polyimide wafers maximized mechanical stability and insured the fidelity of internal electrical connections. The electrode assemblies employed ultrasonically bonded micro-PCBs as both a mechanical backing and breakout board for metallized leads on the polyimide wafer. These features resulted in a mechanically robust, durable implant with ensured optimal performance during chronic *in vivo* use. Generally, the low-profile MSE assemblies demonstrated consistent performance and longevity following chronic implantation. Progressive motor recovery, assessed via SFI in the operated leg, indicated good tolerance of functional implants.

**Figure 10:**
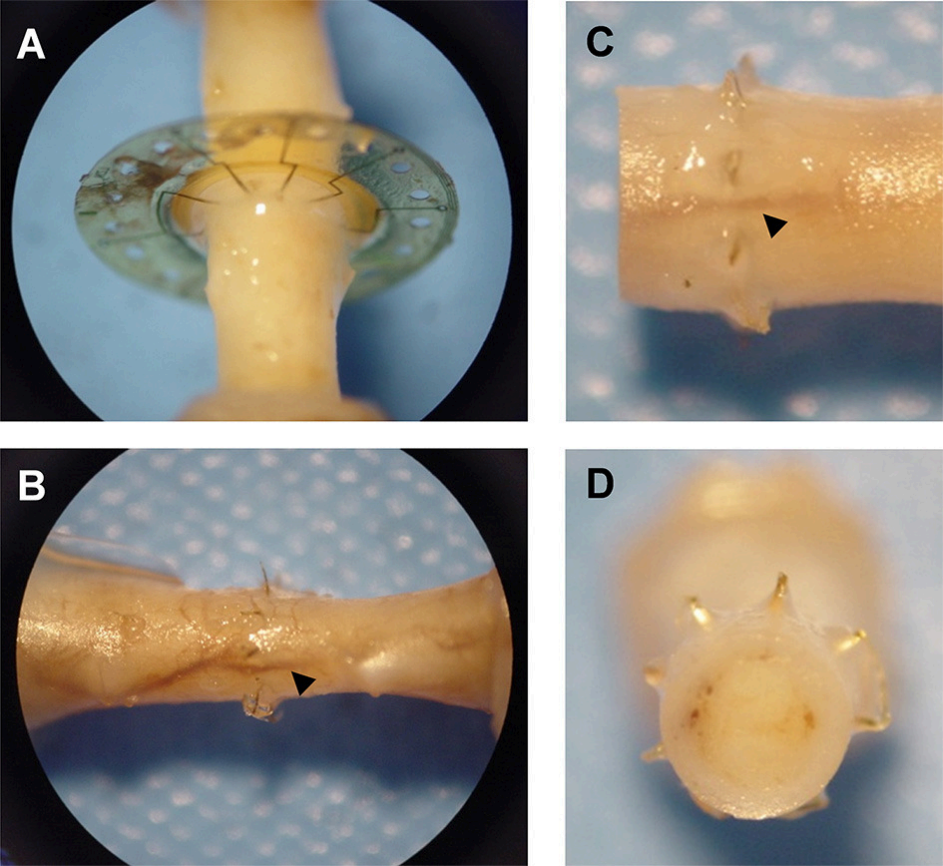
**Integration of macro-sieve electrodes into regenerated nerve tissue**. Photos of regenerated nerve fibers crossing MSEs **(A)** Micro-vasculature (arrowheads) crossing implanted MSE **(B–D)**.

Gross observation of MSEs following long-term implantation confirmed the chronic stability of the assembly and its integration into native peripheral nerve tissue. Dissection and excision of MSEs at terminal time points revealed widespread nerve regeneration through assemblies with and without neurotrophic support. The presence of micro-vasculature integrated within MSE assemblies was notable, along with the absence of any evidence of physical impediment to regenerating nerve fibers (Figure 10).

Histological analysis further confirmed that MSEs supported substantial penetration of regenerated axons through transit zones. We found comparable numbers of regenerated nerve fibers crossing through transit zones in MSEs and through the open lumens of nerve guidance conduits, suggesting minimal impact of MSEs on axonal regeneration post-surgery. Similar to previous studies (Jenq and Coggeshall, 1985; MacKinnon et al., 1991; Archibald et al., 1995), fiber counts within the conduits were significantly more numerous compared to controls. Previous work has also shown that fiber counts proximal and distal to a transection site reach a maximum around 3–6 months following transection (our time of evaluation) and return to control values at 24 months (MacKinnon et al., 1991). Thus, the increased counts observed apparently reflected short-term regeneration following nerve transections and were not a special consequence of axonal regrowth through the implants.

### Functional regeneration through macro-sieve electrodes

Electrophysiological recordings of CNAPs revealed the functionality of regenerated nerve fibers crossing implanted MSEs and nerve guidance conduits, further validating the capabilities of the MSE. Moreover, EMG measurements from distal musculature upon stimulation proximal to implants provided evidence that motor axons regenerated through implanted MSEs and successfully reinnervated multiple muscles without impediment. The critical nature of maintaining distal musculature long after device implantation suggests that MSEs possess significant clinical utility. Selective evoked muscle force measurements further highlight the role of electrode geometry on terminal motor function.

Even in the absence of neurotrophic support, MSEs enabled comparable motor recovery to that found with nerve guidance conduits. The significant impact of the MSE's geometry on motor recovery is notable. These results further confirm the finding that MSEs effectively preclude any physical impediment to regenerating axons, and thereby nerve regeneration. While present studies have not examined the long term (>12 mo) chronic effect of MSEs implantation, observations of equivalent motor function following MSE and silicone nerve conduit implantation suggest that the presence of the high-accessibility transit zones in the polyimide wafer has a negligible effect on regenerating axonal populations. These findings support the conclusion that MSEs designed to facilitate electrical stimulation of motor axons must possess a permissive geometry containing a highly porous region in order to support large populations of regenerating motor axons needed to preserve distal muscle targets.

Stimulation of regenerated nerve fibers using epineurial wire electrodes or stimulation of all MSE electrode sites simultaneously evoked comparable maximum isometric twitch forces; thus, the eight large electrodes were capable of stimulating all axons within the regenerated nerve. Micro-sieve technology utilizes much smaller pore sizes (40–65 μm in diameter vs. 600 μm in MSE) yielding a large number of transit holes per electrode (200–300; Navarro et al., 1998). Given instrumentation limitations, only a subset of micro-sieve transit holes can be surrounded by small active electrodes (8–16 sites); thus, the micro-sieve is not designed to activate all axons within the nerve.

Stimulation of individual electrode sites in MSEs resulted in minute twitch responses evoked at low stimulus amplitudes. The temporal characteristics of evoked force traces did not differ between epineurial and MSE stimulation. However, neither epineurial nor MSE stimulation elicited forces equaling that in controls, indicating possible combinations of muscle atrophy, smaller motor units, and fewer innervating fibers following nerve transection and regeneration. The high recruitment selectivity achieved during monopolar stimulation using individual electrode sites on the MSE suggests preserved spatial clustering of functionally related axons in the transected nerve despite the disruption of normal fasciculation observed in previous studies (Lago and Navarro, 2006). We believe that the quantification of this preservation of spatial clustering may be an interesting line of inquiry for future studies.

### Reduced dependence on neurotropic support

The quality and morphology of regenerated nerve fibers crossing implanted MSEs was comparable in the presence or absence of the GDNF-loaded delivery system. Specifically, nerve histology illustrated that inclusion of GDNF did not have a profound impact on axonal regeneration through implanted MSEs and was not required to establish a sufficient interface between regenerated nerve and electrode sites. While these observations do not negate the importance of neurotrophic support in axonal/nerve regeneration, they suggest that an optimally designed MSE containing highly porous transit zones may reduce the need for neurotrophic support of axonal regeneration.

Results from electrophysiological and muscle force measurements also indicated only minor differences with neurotrophic support in implanted MSE assemblies. GDNF moderately improved CNAP amplitudes, but was not critical to establishing successful motor axon regeneration through implanted electrodes. These observations possibly demonstrate the comparative effects of physical barriers vs. biological cues in axonal regeneration. Particularly, these studies confirm that the design and geometry of implanted electrodes affect total axonal regeneration to a much greater degree than local neurotrophic factors. While neurotrophic support improves and accelerates axonal regeneration through implanted devices, optimized electrode sieve geometry enables sufficient axonal regeneration and motor recovery. Interestingly, the choice of animal model in the present study may influence these observations. Rat sciatic nerve has exceptional neuroregenerative capacity even in the presence of critical nerve injuries and defects (MacKinnon et al., 1985). Thus, the use of neurotrophic support with sieve electrodes designed for human use may provide additional clinical benefit beyond the results of the present study.

## Conclusions

The present work described the manufacture, assembly, and implantation of MSEs with highly porous transit zones much larger (~0.285 mm^2^) than previously tested with micro-sieve electrodes (~1260 μm^2^; Lago et al., 2007). Histomorphometry of regenerated nerve tissue, muscle electrophysiology, and motor functional behavior measurements indicated comparable regeneration through MSEs and empty nerve conduits, showing that MSEs did not impede motor fiber regeneration. The effects of GDNF were moderate suggesting that axon regeneration through the large transit zones in the MSEs was less dependent on neurotropic support compared to micro-sieve electrodes. Electrical monopolar stimulation of different electrode sites within a MSE recruited specific re-innervated muscles, thus indicating the potential of a chronically implanted MSE for functional neuromuscular stimulation of a paralyzed limb.

## Author contributions

MM, JW, and DM designed the macro-sieve electrode. MM and DM designed the experiment. MM and EZ performed the experiment. MM, EZ, and HB analyzed the data. MM, EZ, JW, HB, and DM wrote and edited the manuscript.

## Funding

Portions of this work were sponsored by the Defense Advanced Research Projects Agency (DARPA) Biological Technologies Office (BTO) Hand Proprioception and Touch Interfaces (HAPTIX) program under the auspices of Dr. Doug Weber through the DARPA Contracts Management Office Cooperative Agreement No. HR0011-15-2-0007.

### Conflict of interest statement

The authors declare that the research was conducted in the absence of any commercial or financial relationships that could be construed as a potential conflict of interest.

## Footnotes

^1^Fibrin matrices containing the affinity-based delivery system were prepared in the laboratory of Dr. Shelly Sakiyama–Elbert at Washington University (St. Louis, MO) (Lee et al., 2003).
